# South African Regulatory Authority: The Impact of Reliance on the Review Process Leading to Improved Patient Access

**DOI:** 10.3389/fphar.2021.699063

**Published:** 2021-07-23

**Authors:** Andrea Keyter, Sam Salek, Lorraine Danks, Portia Nkambule, Boitumelo Semete-Makokotlela, Stuart Walker

**Affiliations:** ^1^ Department of Clinical, Pharmaceutical and Biological Sciences, School of Life and Medical Sciences, University of Hertfordshire, Hatfield, United Kingdom; ^2^ Institute for Medicines Development, Cardiff, United Kingdom; ^3^ South African Health Products Regulatory Authority, Pretoria, South Africa; ^4^ University of the Witwatersrand, Johannesburg, South Africa; ^5^ Centre for Innovation in Regulatory Science, London, United Kingdom

**Keywords:** south african regulatory review times, south african health products regulatory authority, backlog, good review practices, regulatory performance

## Abstract

**Background:** The aims of this study were to compare the overall regulatory review timelines achieved by the South African Health Products Regulatory Authority (SAHPRA) in 2020 to the timelines historically achieved by the Medicines Control Council (MCC). This study also aimed to evaluate the regulatory review processes and the good review practices that have been implemented by SAHPRA to support the assessment of new chemical entities and generic product applications for market authorization in the business-as-usual and backlog process streams.

**Methods:** A questionnaire was completed and verified by SAHPRA to describe the structure of the organization, the resources available, the process for regulatory review of new chemical entities and generic products and the level of implementation of good review practices and regulatory decision-making practices for market authorization. Data were collected and analyzed on the overall approval timelines for new chemical entities and generic products registered by SAHPRA in 2020 in the business-as-usual and backlog process streams.

**Results:** A full, independent scientific review was conducted for all new chemical entities and generic product applications in the business-as-usual stream. Facilitated regulatory pathways were introduced for the review of new chemical entities and generic products in the backlog stream. As a result, the timelines for approval of applications in the backlog stream were 68% quicker for both new chemical entities and generics, using facilitated regulatory pathways, such as abridged and verification review models.

**Conclusion:** The comparisons made through this study provided insight into the improvements that have been made through the establishment of SAHPRA and the transition in 2018 from the MCC. The re-engineered processes that have been developed and implemented by SAHPRA to address the backlog in the review of the applications for market authorization have demonstrated a decrease in the overall median approval times. The expansion of these processes into the routine review of medical products will contribute to the enhanced regulatory performance of SAHPRA and patients’ access to new medicines.

## Introduction

The South African Health Products Regulatory Authority (SAHPRA) is the national regulatory authority (NRA) of South Africa, mandated through the Medicines and Related Substances Control Act, 1965 (Act 101 of 1965) to ensure the safety, quality and efficacy of medical products (Republic of South Africa, 2017). In 2017, amendments to Act 101 of 1965, set forth a significant change in the legislature and the organizational structure of the South African NRA and triggered the establishment of SAHPRA to replace the former medicine regulatory authority the Medicines Control Council (MCC) (Keyter et al., 2018a). Historically the MCC had a reputation for robust evidentiary assessments of medical products for market authorization, but was affected by operational challenges and resource constraints that resulted in extended timelines for regulatory review. Efforts were made to reorganize processes and improve the capacity of assessment teams and pilot programs endeavoured to address these issues, but a significant backlog developed, and patients’ access to medicines was delayed.

The SAHPRA has quantified the backlog (BL) and firm targets have been set for its clearance. Inherited processes and practices have been reassessed and pilot projects have been initiated to support new methodologies required to achieve this goal. These include enhanced document management systems, reformed work streams with resources to support this, revised performance contract models, project management tools and the application of facilitated regulatory pathways (FRPs). Applications received by the MCC and/or SAHPRA prior to February 1, 2018 are considered to be part of the BL and have been processed as part of the pilot of the new methodologies. Applications for market authorization that were received by SAHPRA from February 1, 2018 are considered to be in the “business as usual” (BAU) work stream. These applications have been processed using the inherited regulatory review process that was initially developed by the MCC. Keyter and colleagues studied the regulatory review process of the MCC and the overall approval timelines achieved, using this process during the period 2015–2017 (Keyter et al., 2018a). The outcomes of this study serve as a baseline for assessing the changes that have been implemented by SAHPRA as it moves toward enhanced regulatory responsiveness and improved regulatory performance.

National regulatory authorities globally recognize the need to develop and maintain effective and efficient regulatory review processes while managing capacity constraints, legislative limitations, advancing technologies, and large volumes of applications for the market authorization of medical products (Hill and Johnson, 2004; Cone and Walker, 2005; Cone and McAuslane, 2006). Several strategies have been enlisted by NRAs to support regulatory reform. The formal implementation of good review practices (GRevPs) provides the fundamental foundation for regulatory review processes and is a measure on which the regulatory performance of the regulator may be measured. The 10 key principles of GRevPs support consistent, thorough, well-documented, evidence-based, efficient, and well-managed reviews (World Health Organization, 2015). NRAs may also consider the use of FRPs such as reliance mechanisms to conserve limited resources and avoid duplication of regulatory effort. The application of FRPs contribute towards decreased timelines for the evaluation of applications for market authorization as the NRA may rely on or recognize the regulatory decision made by a reference NRA. In this way, the NRA does not need to conduct a full review of the data submitted to support the application for market authorization and can realistically achieve shorter overall approval timelines. With the advent of SAHPRA, opportunities to re-engineer the regulatory review process brought renewed goals for enhanced regulatory performance (Keyter et al., 2018b).

This Study Aimed to

⁃ Evaluate the overall approval timelines for new chemical entities (NCEs) over the period 2015–2020 wherein different review pathways were applied;
⁃ Compare the overall regulatory review timelines achieved by SAHPRA (2018–2020) to the timelines historically achieved by the MCC (2015–2017);
⁃ Compare the South African regulatory review processes that have been implemented by SAHPRA to support the assessment of NCEs and generic product applications for market authorization in the BAU and BL streams;
⁃ Evaluate the level of implementation of GRevPs in the regulatory review process by SAHPRA; and
⁃ Review the challenges and opportunities for enhancing the regulatory performance of SAHPRA leading to decreased overall approval timelines for NCEs and generics.

## Methods

### Study Participants

This study provides a comparison of the two regulatory review processes implemented by SAHPRA for the registration of products in 2020. The first regulatory review process is the one that has been applied to applications for market authorization, received by SAHPRA prior to February 1, 2018, which forms part of the BL stream. The second regulatory review process is the one that has been applied to applications for market authorization, received by SAHPRA from February 1, 2018, which forms part of the BAU stream.

### Study Tool and Data Collection Process

The Centre for Innovation in Regulatory Science (CIRS) developed and validated the tool that was used to collect the regulatory system information for SAHPRA (McAuslane et al., 2009). The tool was presented as a five-part questionnaire that was completed by SAHPRA: part 1 provided insight into the organization and structure of SAHPRA and the available resources; part 2 identified the regulatory review model/s applied by SAHPRA; part 3 was used to map the regulatory review process and the targets and milestones within the review process that allowed for analysis of regulatory performance; part 4 identified the GRevPs that were implemented by SAHPRA to build quality into the regulatory review process; and part 5 explored the evidence supporting the implementation of quality decision-making practices (QDMPs) in regulatory decision making for market authorization.

The questionnaire was completed in duplicate to reflect the regulatory review processes that were applied by SAHPRA to applications for market authorization within the BL and BAU streams. The questionnaires were completed by the various unit heads within SAHPRA and verified by the Chief Regulatory Officer and Chief Executive Officer of SAHPRA. Data were collected to reflect the median regulatory review timelines for NCEs (including biologicals) and generics from the date of receipt of the application (prior to February 1, 2018 = BL; after February 1, 2018 = BAU) to the date of approval in 2020. The data were sourced directly from the SAHPRA Units responsible for monitoring and evaluating the regulatory performance metrics. It should be noted that no data were available for 2019, as the agency was unable to operate during this period due to a number of mitigating circumstances.

### Models of Regulatory Review

#### Review Assessment Type I—Verification Model

The verification model, as defined by McAuslane and associates, provides a mechanism for NRAs to rely on or recognize the regulatory decision of a reference NRA (McAuslane et al., 2009). This model promotes faster regulatory review times as the data submitted is verified for compliance against that which was submitted to the reference NRA. The use of the verification model allows the NRA to avoid duplication of regulatory effort and to conserve limited resources.

#### Review Assessment Type II—Abridged Review Model

McAuslane and colleagues describe the abridged review model as a selective review of the data submitted for market authorization, provided that the product has been registered by a reference NRA (McAuslane et al., 2009). This type of review is often limited to an assessment of the country-specific requirements for product quality and the clinical data associated with the benefit-risk assessment of the product.

#### Review Assessment Type III—Full Review Model

The full review model requires a full independent scientific assessment of the data submitted for market authorization (McAuslane et al., 2009). This review model does not require market authorization with a reference NRA as a prerequisite.

### Ethics Approval

The study has been approved by the Health, Science, Engineering and Technology ECDA, University of Hertfordshire [Reference Protocol number: LMS/PGR/UH/03873].

## Results

### Comparative Assessment of Regulatory Review Processes and Milestones

**FIGURE 1:**
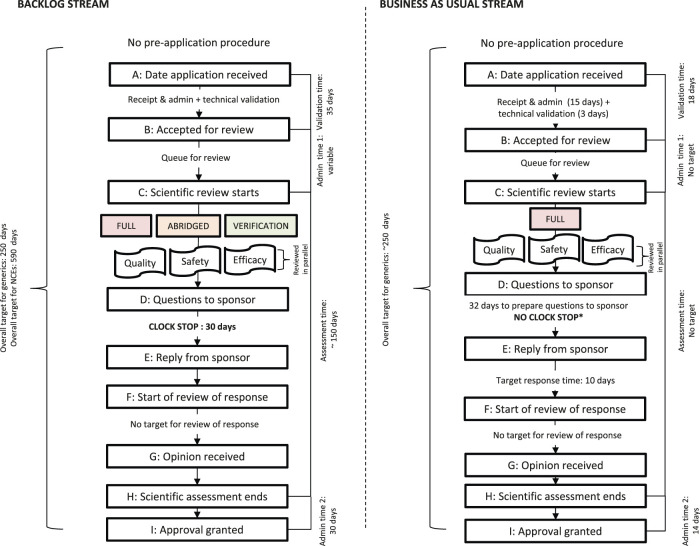
Registration process map for the SAHPRA backlog (BL) stream and business-as-usual (BAU) stream. *No clock stop indicates that the sponsor’s response time is included in the overall review time

Figure 1 shows a simplified illustration comparing the SAHPRA registration review process, milestones, and target review times for the BAU and BL process streams. Figure 1 represents the process for NCEs and generics that were approved in the first review cycle and does not represent the process for applications that were not recommended for market authorization.

The NCE and generic applications for market authorization received prior to February 1, 2018, in the BL stream were processed by SAHPRA using the full review model, the abridged review model or the verification model. Registration with at least one reference NRA was a prerequisite for the use of the abridged review model and registration with at least two NRAs was required for the verification model. SAHPRA required the applicant to provide additional data to support the indications if there were differences in the product indications between the registrations approved by the two reference NRAs. The NCE and generic applications for market authorization, received after February 1, 2018 were assessed in the BAU stream, using the full, independent, scientific review model.

Assessments of applications for market authorization were conducted by both internal and external assessors. SAHPRA appointed a total of 113 assessors to review applications for market authorization, of whom 57 were allocated to review applications for BAU only. The primary scientific assessment was followed by a peer-review, conducted by a second assessor. Expert committees were used in the review process in an advisory capacity. The clinical and quality expert committees were engaged in the BAU review process while only the clinical expert committee participated in the BL review.

Products requiring priority review were “fast-tracked” provided that the priority designation was confirmed at pre-registration through identification by the National Department of Health, to meet an unmet public health need. Products eligible for priority review were usually already registered in another jurisdiction. A copy of the registration certificate was required, although a Certificate of Pharmaceutical Product was not. The data requirements and scientific assessment criteria for priority products were the same as those for other products, but priority review products were not placed in the queue for review and were allocated immediately to an assessor. The target timeline for the review of priority products was 350 calendar days; however, there were no products fast tracked for priority review in the BAU or the BL streams in the data reviewed in this study.

### Data Requirements

The SAHPRA placed reliance on and/or recognized the regulatory decisions of reference NRAs/organizations/collaborative initiatives to inform their own regulatory decision making. The SAHPRA considered the following NRAs as reference agencies: United States Food and Drug Administration (USFDA), the European Medicines Agency (EMA), individual European Union Member States, the Japanese Pharmaceuticals and Medical Devices Agency (PMDA), Health Canada, Swissmedic, the Medicines and Healthcare products Regulatory Agency (MHRA) of the United Kingdom and the Australian Therapeutic Goods Administration (TGA). The SAHPRA also relied on and/or recognized product pre-qualifications performed by the World Health Organization (WHO) and the regulatory decisions made through the Zazibona Collaborative Medicines Registration pathway. The SAHPRA applied reliance pathways in the review of products in the BL stream but did not apply a reliance strategy in the assessment of products in the BAU stream. SAHPRA did not have a formal pre-application procedure in place and there was no requirement for the applicant to submit a notification of intent to make application for market authorization while there was clear published data requirements for the assessment of applications for market authorization using the verification, abridged, and full review models (Table 1).

**TABLE 1:** Data requirements for the review models and extent of the scientific assessment.

Type of review model	Verification model	Abridged review model	Full review model
NCEs + generics in BAU	✗	✗	✓
NCEs + generics in BL	✓	✓	✓
ADDITIONAL INFORMATION OBTAINED			
Registration by reference NRA	✓^a^	✓^b^	✗
Certificate of pharmaceutical product	✓	✗	✗^c^
Similarity to registered product	✓^d^	✓^d^	✗
EXTENT OF SCIENTIFIC ASSESSMENT			
Quality data	✓^e^	✓^e^	✓^f^
Non-clinical data	✓^g^	✓^g^	✓^f^
Clinical data	✓^g^	✓^g^	✓^f^

Adapted from Keyter et al., 2018a.

BAU, business as usual; BL, backlog; NRA, national regulatory authority; NCE new chemical entity.

a Requires registration by at least two reference NRAs.

b Requires registration by at least one reference NRA.

c Required if available at the time of submission.

d The dosage form, strength, ingredients, indications, and dosage, warnings and precautions, product label and product name must be identical to the product registered by the reference NRA.

e A checklist review for completeness of data is conducted.

f A full review is conducted.

g A selective review in detail is conducted.

SAHPRA required the submission of the full common technical document (CTD) to support the application for market authorization, irrespective of the model of assessment applied. The extent of the scientific assessment performed by SAHPRA differed based on the model of assessment applied. For the verification and abridged review models, SAHPRA required the applicant to request the reference NRA to give SAHPRA permission to access the un-redacted assessment report of the registered product. If the un-redacted report was not available, SAHPRA would obtain the redacted assessment report from the public domain. The redacted report would be accepted if the report contained sufficient scientific data, such as the inclusion of information pertaining to the active pharmaceutical ingredient (API), to inform regulatory decision making. The abridged review model was applied with a focus on the use of the product under local conditions. The clinical opinion took account of the national disease patterns, ethnic factors, unmet medical needs, and differences in medical practice and culture. The full review model was further stratified by SAHPRA as either A) whereby a full review was performed and information on prior registration of the product in another jurisdiction was a prerequisite for market authorization; or B) whereby a full, detailed, independent review was performed.

### Target and Approval Times

The transition of the MCC to SAHPRA brought with it an opportunity to re-engineer the South African regulatory review process. The regulatory review processes implemented by SAHPRA for the BAU and BL streams and the associated milestones and timelines are depicted in Figure 1. The milestones within the review process were previously defined (Keyter et al., 2019a). SAHPRA target timelines for the milestones within the BAU and BL regulatory review processes have been compared against the previous MCC target timelines (Table 2). SAHPRA target timelines for validation of the application (35 calendar days) exceeded the MCC target timeline (15 calendar days) and the target timeline for validation of BL applications (35 calendar days) was longer than for BAU applications (18 calendar days). There was no target timeline for scientific assessment for applications in the BAU stream. The target scientific assessment time for applications in the BL (150 calendar days) was longer than the MCC target timeline for scientific assessment (90 calendar days). There were no target timelines for the expert committee review in both the BAU and BL streams. The target timelines for the authorization procedure decreased in comparison to the previous MCC target timeline.

**TABLE 2:** Target timelines for SAHPRA review process compared with previous MCC timelines.

Process	MCC^a^	SAHPRA BAU	SAHPRA BL
Validation	15 calendar days	18 calendar days	35 calendar days
Scientific assessment	90 calendar days	No target	∼150 calendar days
Sponsor response time	180 calendar days	42 calendar days	30 calendar days
Expert committee(s)	60 calendar days	No target	No target
Administration time	60 calendar days	14 calendar days	35 calendar days
Notification of decision	7 calendar days	10 calendar days	7 calendar days
Overall review time (fast track)	250 calendar days	350 calendar days	350 calendar days
Overall review time (NCEs)	No target	590 calendar days	250 calendar days
Overall review time (generics)	No target	250 calendar days	250 calendar days

a Keyter et al., 2018a.

BAU, business as usual; BL, backlog; MCC, Medicines Control Council; NCEs, new chemical entities; SAHPRA, South African Health Products Regulatory Authority.

The median overall approval timeline for NCEs (783 calendar days) decreased by 63% in 2020 compared to the highest median overall approval timeline for NCEs (2,124 calendar days) that was recorded in 2018 (Table 3). This was much shorter than the median timelines for MCC over the three-year period (2015–2017).

**TABLE 3:** Median overall approval timelines for NCEs from 2015–2020.

NRA	MCC^a^	MCC^a^	MCC^a^	SAHPRA^a^	SAHPRA^b^	SAHPRA
Year	2015	2016	2017	2018	2019	2020
Number of NCEs approved	31	33	42	15	—	155
Median overall approval timeline	1,175	1726	1,466	2,124	—	783

MCC, Medicines Control Council; NCE, new chemical entities; NRA, national regulatory authority; SAHPRA, South African Health Products Regulatory Authority.

a Keyter, et al., 2019a.

b No data were available for 2019.

**FIGURE 2:**
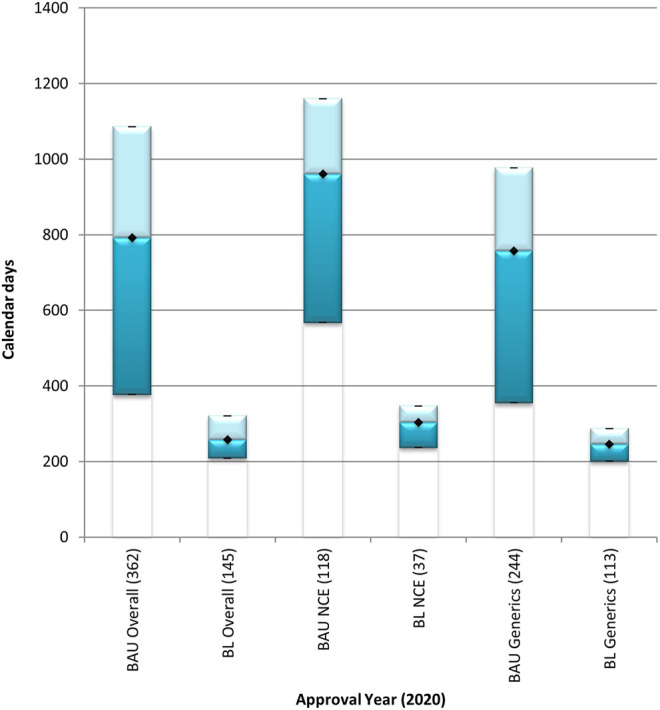
Median overall approval times for new chemical entities and generics processed through the BAU and BL review streams in 2020. BAU, business as usual; BL, backlog; NCE, new chemical entity. Data are shown for NCEs and generics that were approved in 2020 through the business as usual and the backlog process streams. (n) = number of product applications registered in 2020. Box: 25th and 75th percentiles. Whiskers: 5th and 95th percentiles. Denotes median.

The overall median approval timeline for both the NCEs and generic product applications was 792 calendar days in the BAU stream and 257 calendar days in the BL stream in 2020 (Figure 2). The overall approval timeline for NCEs was 961 calendar days and 303 calendar days for the BAU stream and the BL stream, respectively. The time taken to approve NCEs using the FRPs applied in the BL stream was 68% quicker than the time taken to approve NCEs in the BAU stream. The overall approval timeline for generics was 756 calendar days for the BAU stream and 246 calendar days for the BL stream. The overall approval timeline for generics decreased by 68% in the BL stream when compared to the timelines observed for the approval of generics in the BAU stream.

**FIGURE 3:**
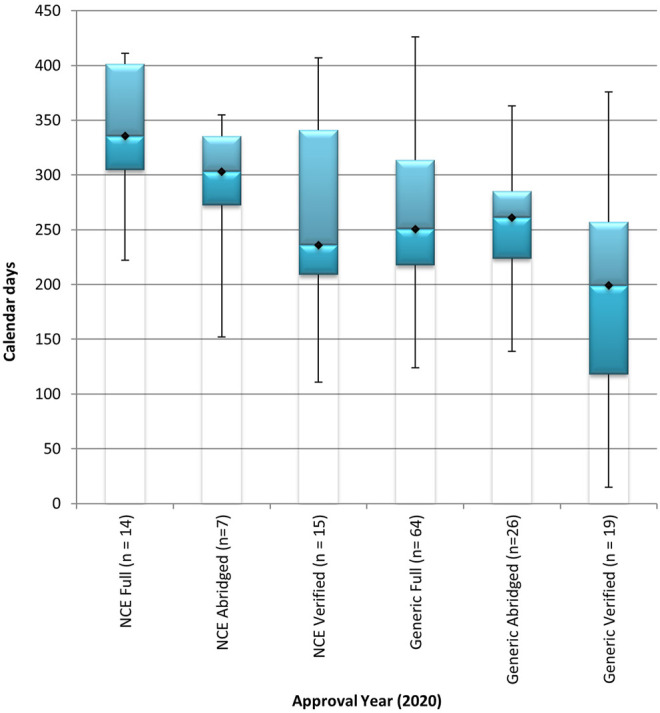
Median overall approval times for new chemical entities and generics, in the backlog review stream, processed through the full review model, the abridged review model, and the verification model in 2020. BL, backlog; NCE, new chemical entity. Data are shown for NCEs and generics in the backlog process stream that were approved in 2020 using the full, abridged and verification review models. (n1) = number of product applications registered in 2020. Box: 25th and 75th percentiles. Whiskers: 5th and 95th percentiles.

The NCEs and generics in the BL review stream that were approved in 2020 were processed through the full review model and through FRPs including the abridged review model and the verification model (Figure 3). The overall median approval timeline for NCEs in the BL stream was 336 calendar days using the full review model, 303 calendar days using the abridged review model, and 236 calendar days using the verification model. The use of FRPs demonstrated a decrease of 10% for abridged review of NCEs and a decrease of 30% for verification of NCEs when compared to the time taken to conduct a full review of NCEs in the BL stream. The overall median approval timeline for generics in the BL stream was 251 calendar days, 261 calendar days, and 199 calendar days, using the full review model, the abridged review model, and the verification model, respectively. The time taken to review a generic application in the BL stream, using the verification model was 21% less than the time taken using the full review model. An increase of 4% was observed in the application of the abridged review model when compared to the time taken to conduct a full review of generics in the BL stream.

### Comparative Assessment of Good Review Practices

The status of the implementation of GRevPs by the MCC was evaluated to determine the effectiveness of the regulatory review process (Keyter et al., 2018a). Since the inception of SAHPRA, further work has been done to formalize the implementation of the GRevPs in an effort to enhance the regulatory review process. Table 4 provides a comparison of the implementation of the parameters of the GRevPs that were implemented by the MCC and the progress that has been made by SAHPRA.

**TABLE 4:** Status of implementation of good review practices by SAHPRA.

Indicator	MCC status^a^		Comments^a^	SAHPRA status		Comments
Quality measures						
Internal quality policy	✓		Planned to formally implement	✓		Implemented
Good review practice system	✓		Planned to formally implement	✓		Improvement required to support timeliness of review
Standard operating procedures (SOPs) for guidance of assessors	✓		Planned to formally implement	✓		SOPs for all procedures in the regulatory review to be formalized
Assessment templates	✓		Planned to formalize the use of a single, common template	✓		Templates for clinical assessment and abridged and verification reviews have been formally implemented
Dedicated quality department	✗		Establishment of a dedicated quality department is planned	✓		Quality manager has been appointed, team to be recruited
Scientific committee	✓		—	✓		—
Shared and joint reviews	✓		—	✓		—
Transparency and communication parameters						
Feedback to industry on submitted dossiers	✓		—	✓		—
Details of technical staff to contact	✓		Contact details are made available on an ad-hoc basis	✓		Contact details are made available on an ad-hoc basis
Pre-submission scientific advice to industry	✓		Meetings are held with industry on an ad-hoc basis	✓		Pre-submission advice is provided for only biologicals and various COVID-19 related applications
Official guidelines to assist industry	✓		—	✓		—
Industry can track progress of applications	✗		Implementation of electronic document management system is planned	✓		Tracking was implemented for applications in the backlog and BAU process streams
Publicly available summary basis of approval (SBA)	✗		Summary is available but is currently not published	✗		ZAPAR not produced or published but planned to implement in future
Approval times	✓		Approval times are not made available to the public	✓		Approval times are not made available to the public
Advisory committee meeting dates	✓		—	✓		—
Approval of products	✓		—	✓		—
Continuous improvement initiatives						
External quality audits	✓		External quality audits are not performed routinely	✓		External quality audits are now in place
Internal quality audits	✗		Planned	✓		Planned to be formally implemented and conducted by the quality department
Internal tracking systems	✓		Implementation of electronic document management system is planned	✓		Tracking implemented for applications in the backlog and BAU process streams
Review of assessors’ feedback	✓		—	✓		—
Reviews of stakeholders’ feedback	✓		Planned to be formally and routinely reviewed	✓		Formally implemented
Training and education						
International workshops/conferences	✓		—	✓		—
External courses	✓		—	✓		—
In-house courses	✓		Training program to be formalized	✓		Training program to be formalized and effectiveness of training to be measured
On-the-job training	✓		Training program to be formalized	✓		Training program to be formalized and effectiveness of training to be measured
External speakers invited to the authority	✓		—	✓		—
Induction training	✓		Training program to be formalized	✓		Training program to be formalized and effectiveness of training to be measured
Sponsorship of post-graduate degrees	✓		—	✓		—
Placements and secondments in other regulatory authorities	✓		—	✓		—

Legend		Formally implemented		Informally implemented		Not implemented	

a Adapted from Keyter et al., 2018a.

MCC, Medicines Control Council; SAHPRA, South African Health Products Regulatory Authority; SBA, Summary Basis of Approval; SOP, standard operating procedure, ZAPAR, South African public assessment report.

### Enablers and Barriers to Good Quality Decision Making

**FIGURE 4:**
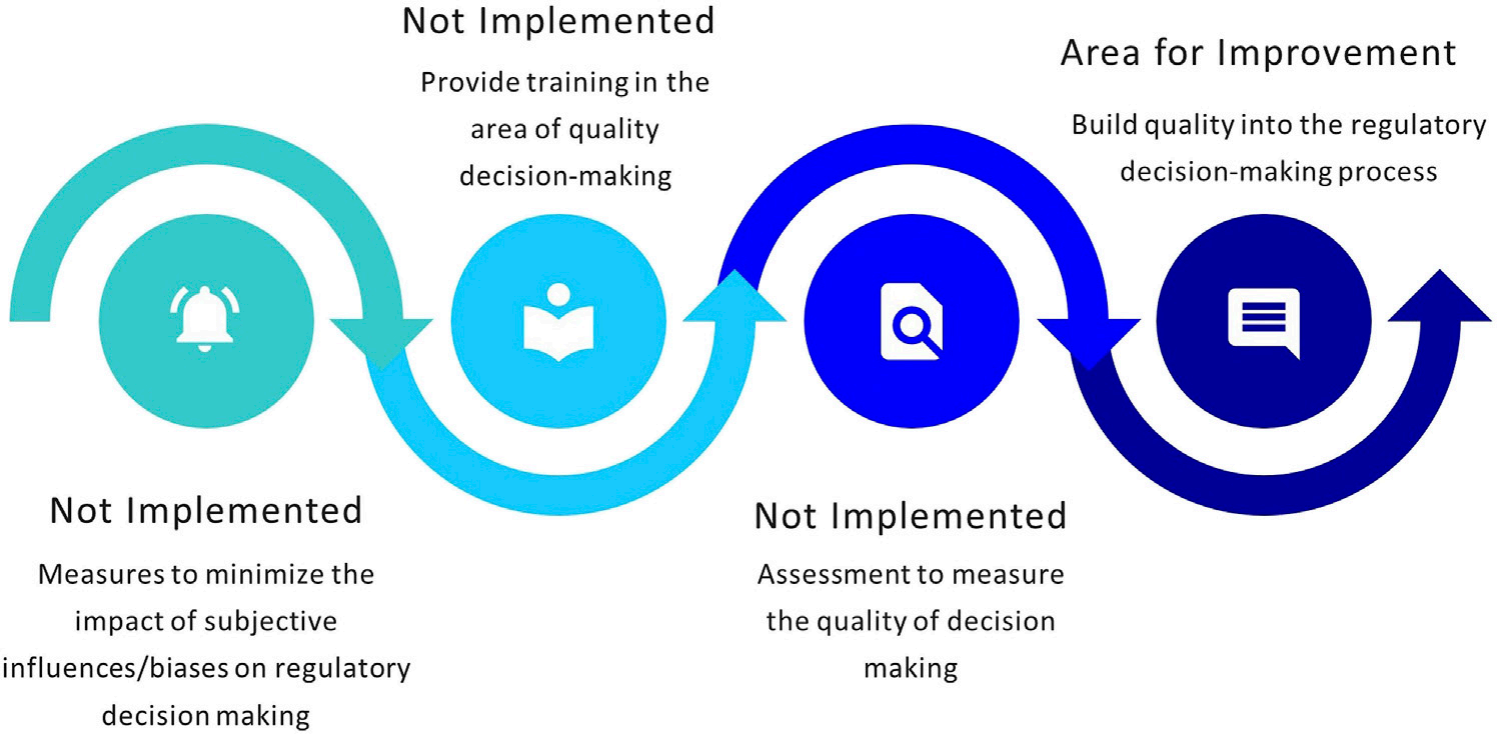
Status of implementation of quality decision-making practices.

The MCC developed a framework for regulatory decision making based upon the formalized legislature, policies, and guidelines. The study conducted by Keyter and colleagues did not provide an assessment of the QDMPs considered and/or implemented by the MCC (Keyter et al., 2018a). SAHPRA has adopted the regulatory decision-making framework established by the MCC; however, the process of building quality into decision-making practices has not been formalized. The agency assessed which of the 10 QDMPs have been implemented (Bujar et al., 2017) and the outcome of this process is illustrated in Figure 4.

## Discussion

### Review Type and Process

Globally, NRAs have shifted their focus toward the enhancement of regulatory performance and faster regulatory approval timelines. The advent of the implementation of FRPs allows NRAs to rely on or recognize the regulatory decisions of other reference agencies. Making use of the abridged review model and the verification model allows NRAs to decrease the time taken to review an application for market authorization (Keyter et al., 2020a). SAHPRA has introduced the use of the abridged review and verification models to the applications in the BL stream. Generally, the results demonstrated a decrease in the time spent on the review of applications that were channelled through FRPs.

In this study, the time taken to perform a verification was significantly less than the time taken to perform a full review. It is interesting that the review time for generics that underwent the abridged review took longer than those that underwent the full review. It is likely that the extent of the review conducted using the abridged review model has not been sufficiently modified to a selective, detailed review, limited to specific parts of the submitted CTD. It was noted that the verification model applied by SAHPRA currently requires a specific detailed review of non-clinical and clinical data; and that applications were routed to ensure that the product submitted to SAHPRA was identical to the product registered by the reference NRA/s. These practices may have contributed to the extended validation period (increased from 15 calendar days to 35 calendar days) identified for products in the BL stream that were subjected to verification and abridged review. In line with the definitions for reviews using the verification model or the abridged review model (McAuslane et al., 2009), it would be prudent to limit reviews using the verification model to a check for completeness of data instead of a selective, detailed review; and accepting the signed declaration of sameness from the applicant without conducting an additional cross-check to confirm sameness for each part of the CTD submitted. Routine implementation of these practices will ensure that limited resources are conserved, that the regulatory effort is commensurate with the level of risk of the product, and that the assessors are able to consistently exercise the level of restraint required for the extent of the review that is recommended when performing a review using the verification model or the abridged review model (Keyter et al., 2020a).

### Target and Approval Times

The overall median approval timeline for medical products, achieved by an NRA, is largely indicative of the regulatory performance of the NRA. In order to enhance regulatory performance, NRAs are recommended to monitor and evaluate their regulatory performance metrics. This can be achieved by documenting the regulatory review process, identifying the milestones within the process, and determining the target timelines to be achieved for each activity. The review process that had been developed and implemented by the MCC was adopted by SAHPRA, upon its establishment as the new South African NRA in 2018 (Keyter et al., 2019b). Data reflecting the overall approval timelines for NCEs, achieved by the MCC from 2015–2017 and by SAHPRA in 2018, demonstrated extended timelines and unacceptable regulatory performance that negatively affected patients’ access to medicines in South Africa (Keyter et al., 2019b). The overall approval timeline for NCEs has significantly decreased since 2018 and this result is a testament to the positive impact of the changes that have already been introduced by SAHPRA. The overall approval timelines for applications in the BL are significantly less than that achieved for the applications in the BAU stream. It is acknowledged that most of the products approved in 2020 were part of the BAU stream; however, the FRPs, operational processes, additional infrastructure, and formalization of the performance contracts, the assessment guidelines, and the templates have been pivotal in accelerating the review of the applications in the BL stream.

Historically, the target timelines set by the MCC were not achievable and the legislative and operational influences that negatively affected the performance of the MCC have been previously reported (Keyter et al., 2018b). SAHPRA has identified target overall approval timelines for the review of NCEs and generics in the BL and an overall target timeline for all products in the BAU stream. The target overall approval timelines identified for BAU (250 calendar days) are shorter than those identified for priority-review products (350 calendar days). Currently, the median overall approval timeline achieved by SAHPRA for BAU is 792 calendar days, more than three times the current target. Mature regulatory authorities such as the TGA, Health Canada, and the HSA have set targets for NCE approval at 305 calendar days, 355 calendar days, and 270 working days, respectively. This may indicate that the targets that have been set by SAHPRA have been ambitious, and that if these targets are to be formally adopted as key performance indicators, dedicated efforts will be required to meet these goals.

### Good Review Practices

NRAs are mandated to ensure the safety, quality, and efficacy of medical products. NRAs review applications for market authorization against scientific and evidentiary requirements to inform regulatory decision making to approve or refuse the registration of a product. The NRAs are required to effect their regulatory mandate in a timely manner (World Health Organization, 2015). They can enhance their regulatory systems by incorporating GRevPs in routine regulatory undertakings and ultimately improve their regulatory performance (World Health Organization, 2014a; World Health Organization, 2015). SAHPRA has worked diligently to formalize the implementation of GRevPs over the last 3 years; however, there are two areas for improvement that have been identified. First, a key feature of the GRevPs is a well-documented and thorough report of the scientific data assessed to support the documented rationale for the regulatory decision. SAHPRA should consider the implementation of the Universal Model for Benefit-Risk Assessment (UMBRA) framework as a template that may be used for the clinical assessment informing the benefit-risk decision (Keyter et al., 2020b, Walker et al., 2011; Walker et al., 2013). The UMBRA framework has been validated and utilized by many mature NRAs (Walker et al., 2011) and those in emerging markets (Mashaki Ceyhan et al., 2018). Second, SAHPRA should recognize the importance of a well-managed review as a leading principle of GRevPs. In this regard it is expected that a good review is supported by a fully integrated project management process and a quality management process underpinned by defined target timelines and achievable performance metrics (World Health Organization, 2015). As such, setting achievable targets and routinely measuring and evaluating performance metrics will serve to further enhance and continuously improve the regulatory output and responsiveness of SAHPRA.

SAHPRA has appointed a quality manager and is in the process of further capacitating a Quality Department. The additional resources in the Quality Department will provide the foundation for the codification of the internal quality policy, the planned routine internal auditing activities, and the sustainability of the formalized implementation of a robust quality management system. As a result, opportunities exist for SAHPRA to develop and implement standard operating procedures for each of the regulatory review processes, update templates for clinical assessment to elucidate the benefit-risk assessment in a structured, systematic documented way, and build QDMPs into the review (Keyter et al., 2020b).

SAHPRA does not publish the summary basis of approval that documents the benefit-risk decision; however, the decision to recommend or not recommend a product for market authorization is communicated to the applicant. SAHPRA is considering the development and publication of the South African public assessment report (ZAPAR) to ensure consistency and enhance transparency in regulatory decision making (Keyter et al., 2020b). The routine publication of the ZAPAR may serve to promote the position of SAHPRA as a reference NRA, whose regulatory decisions may be relied upon or recognized by other NRAs.

SAHPRA has recognized the importance of implementing an electronic document management system (EDMS) that is driven by integrated and adequate information and communication technology infrastructure, to facilitate effective project management and tracking of applications for market authorization (Hill and Johnson, 2004; Keyter et al., 2018b). The opportunity to pilot the EDMS has been harnessed by SAHPRA in its application within the BL process stream. The pilot implementation of the EDMS has facilitated internal and external tracking of documents; ultimately contributing to increased transparency and enhanced trust from both internal and external stakeholders.

SAHPRA has implemented several training and education modalities (Keyter et al., 2018a); however, the formalization of the training programs required to support induction training, on-the-job training, and in-house courses has not been formally documented and encoded into organization-wide training structures. As SAHPRA moves forward with this activity it will be critical to incorporate a formalized, robust mechanism to implement, evaluate, and evaluate the effectiveness of training activities (World Health Organization, 2018a).

### Quality Decision-Making Practices

SAHPRA has adopted the historic framework for regulatory decision-making developed by the MCC. A systematic, structured approach to decision making is in place and the roles and responsibilities of assessors and committee members are well defined. A basic record trail of decisions made is available and decisions are re-evaluated in the event of new information becoming available. NRAs are required to demonstrate the effective application of QDMPs (World Health Organization, 2018a). While there is a robust policy for the management of potential or identified conflicts of interest, SAHPRA does not currently evaluate and document internal and external influences and biases, as well as heuristics and uncertainties that may exist. It is recommended that QDMPs are evaluated using the Quality Decision Orientation Scheme (QoDoS) (Donelan et al., 2016; Bujar et al., 2017). Building quality into regulatory decision-making practices would contribute towards well-documented, clear and succinct decisions, that are consistent and transparent in terms of the criteria for registration, that cannot be contested, and that could potentially be relied on or recognized by others (Keyter et al., 2020c; World Health Organization, 2018b).

### Recommendations

The historical operations of the MCC have been compared with the processes that have been reengineered by SAHPRA to address the applications for market authorization in the BL, as well as the routine applications received by SAHPRA in the BAU stream. SAHPRA has made notable strides towards achieving their goal for enhanced regulatory performance. Many changes and improvements have been implemented under the leadership of the new CEO of SAHPRA and the supporting management team. While the strengths of SAHPRA have been identified, it is important to recognize areas for further improvements. Based on the outcomes of this study, the following recommendations should be considered.

⁃ **Reliance strategy:** Implementation of facilitated regulatory pathways for all applications for market authorizations and implementation of a risk-based approach to the regulatory review to ensure that; the specific parts of the CTD to be reviewed are identified, the extent of the review is limited proportionately to product risk; and the application of risk-based regulatory review practices is consistent
⁃ **Benefit-risk assessment:** Implementation of a benefit-risk assessment framework such as the UMBRA in the regulatory process
⁃ **Target timelines:** Establish and enforce achievable target timelines for the regulatory review process and routinely and accurately measure and evaluate regulatory performance metrics
⁃ **Public assessment reports:** Consider the development and publication of the ZAPAR to ensure consistency and transparency in regulatory decision making and to endorse the status of SAHPRA as a potential reference NRA
⁃ **Quality decision-making practices:** Consider the integration of QDMPs to support consistent, evidence-based, transparent practices that support the publication of regulatory decisions in the public domain.

### Limitations of the Study

The lack of data for the year 2019 could be considered as a limitation of this study. The mitigating circumstances were that the former SAHPRA premises were declared unfit as a working environment, which necessitated a major move to new accommodations. As a result, no products were reviewed in that period.

## Data Availability

Raw data cannot be made available due to the nature of the study.

## Abbreviations

API, active pharmaceutical ingredient; BAU, business as usual; BL, backlog; CEO, Chief Executive Officer; CRO, Chief Regulatory Officer; CMC, chemistry, manufacturing and control; CPP, certificate of pharmaceutical product; CTD, Common Technical Document; EDMS, electronic document management system; EMA, European Medicines Agency; FRPs, facilitated regulatory pathways; GMP, good manufacturing practice; GRevPs, good review practices; MCC, Medicines Control Council; MHRA, Medicines and Healthcare products Regulatory Agency; MLE, major line extension; NCEs, new chemical entity; NRA, national regulatory authority; PIC/S, Pharmaceutical Inspection Co-operation Scheme; PMDA, Pharmaceuticals and Medical Devices Agency; SAHPRA, South African Health Products Regulatory Authority; TGA, Therapeutic Goods Administration; UMBRA, Universal Model for Benefit-Risk Assessment; US FDA, United States Food and Drug Administration; WHO, World Health Organization
